# Acute malnutrition relapse and associated factors among 6–59 months old children treated in the community-based management of acute malnutrition in Dessie, Kombolcha, and Haik towns, Northeast Ethiopia

**DOI:** 10.3389/fpubh.2023.1273594

**Published:** 2024-01-08

**Authors:** Yibeltal Asmamaw Yitayew, Zemen Mengesha Yalew, Samuel Nebiyu, Desalegn Abebaw Jember

**Affiliations:** ^1^Department of Pediatric and Child Health Nursing, College of Medicine and Health Science, Wollo University, Dessie, Ethiopia; ^2^Department of Comprehensive Nursing, College of Medicine and Health Science, Wollo University, Dessie, Ethiopia; ^3^Department of Pediatric Nursing, St. Paul Millennium Medical College, Addis Ababa, Ethiopia

**Keywords:** relapse, acute malnutrition, CMAM, under-five, associated factors, Ethiopia

## Abstract

**Introduction:**

Undernutrition is a major health concern in many developing countries, and is one of the main health problems affecting children in Ethiopia. Although many children experience multiple relapses following the management of severe acute malnutrition, it is scarcely studied in Ethiopia.

**Methods:**

A community-based cross-sectional study was conducted in Dessie, Kombolcha, and Haik towns among 6-59-month-old children enrolled and discharged from community-based acute malnutrition management (CMAM). The total sample size was 318 children, and data were collected from April 15, 2021, to May 14, 2021. The data were entered into EPI data version 4.4.1 before being exported and analyzed with SPSS version 25 software. A multivariate logistic regression analysis was performed, and a 95% confidence interval and *p*-value <0.05 were used to identify significantly associated variables. Additionally, the weight-for-height z-score (WHZ) was generated using the WHO Anthro 3.2.2 software.

**Result:**

The overall acute malnutrition relapse after discharge from CMAM was 35.2% (6.6% relapsed to severe acute malnutrition and 28.6% relapsed to moderate acute malnutrition). The following variables were significantly associated with the relapse of acute malnutrition: child age (AOR: 3.08, 95% CI; 1.76, 5.39), diarrhea after discharge (AOR: 2.93, 95%CI; 1.51, 5.69), have not immunized (AOR: 3.05, 95% CI; 1.14, 8.23), MUAC at discharge (AOR: 3.16, 95% CI; 1.56, 6.40), and poorest and poor wealth index (AOR: 3.65, 95% CI; 1.45, 9.18) and (AOR: 2.73, 95% CI; 1.13, 6.59), respectively.

**Conclusion:**

Over one-third of children treated with the CMAM program reverted to SAM or MAM. The age of the child, diarrhea after discharge, lack of immunization, MUAC at discharge (<13 cm), and poor and poorest wealth index were significantly associated with acute malnutrition relapse. Therefore, adequate health education and counseling services are essential for mothers to improve child immunization coverage and maintain adequate hygiene to prevent diarrhea. In addition, further experimental research is needed to investigate the effect of MUAC at discharge on the risk of acute malnutrition relapse.

## 1 Introduction

Malnutrition in children refers to deficiencies, excesses, or imbalances in the nutrient intake. It addresses three groups of conditions: undernutrition, micronutrient-related malnutrition, and overweight or obesity (1). Malnutrition in children can be either acute (wasting) or chronic (stunting). Based on severity, acute malnutrition can be either moderate acute malnutrition (MAM) or severe acute malnutrition (SAM) (2, 3). Children with SAM are treated either in an outpatient therapeutic program or inpatient setting based on the age of the child, severity of malnutrition and the presence or absence of complications. Subsequently, they will be discharged from the therapeutic program when their weight-for-height/length (WHZ) is > − 2 Z-score, mid-upper-arm circumference (MUAC) is ≥12.5 cm, and there is no edema for at least 2 weeks (4).

Severe acute malnutrition is one of the most common causes of morbidity and mortality among children under 5 years of age worldwide. Children with SAM are 10 times more likely to die than non-malnourished children (5). According to the 2019 estimate, globally, 144 million under-5-year-old children were stunted, 47 million were wasted, and 38 million were overweight. Approximately 45% of deaths among children under 5 years of age are linked to undernutrition (1, 6, 7). Undernutrition is a major health concern in many developing countries, and is one of the main health problems affecting children in Ethiopia. Ethiopia has the second-highest malnutrition rate in sub-Saharan Africa (8). According to the 2016 Ethiopian Demographic and Health Survey, 37% of children under age 5 were stunted, 10% were wasted, 24% were underweight, and 1% were overweight (9).

Relapse after treatment for acute malnutrition is a serious health problem faced by humanitarian and developmental communities (10). Many children experience multiple relapses, and relapsed cases take longer to recover (11). Studies indicate that nearly half of all children who are successfully treated for malnutrition do not maintain their recovery for up to 12 months (12, 13). This situation undermines the cost-effectiveness of community-based management of acute malnutrition (CMAM) treatment programs by deploying limited resources to treat the same child multiple times. Furthermore, repeated episodes of severe acute malnutrition (SAM) increase the risk of death and long-term developmental problems and contributing to a persistently high rate of malnutrition (14).

Globally, the magnitude of malnutrition relapse ranges from 0 to 37%, and in Ethiopia, the re-admission (relapse) rate ranges from 2.6 to 37.5% (15–20). The risk of relapse or death was highest in the 3 months following release from the CMAM, with nearly half of all relapses to either moderate acute malnutrition or SAM occurring during the first 3 months of recovery (11). Child feeding practices, sex, food insecurity, lack of vitamin A supplements, prelacteal feeding, MUAC at discharge, complete vaccination, lower anthropometric measurements, and poor linear growth are the main factors contributing to the relapse of acute malnutrition (13, 21–23).

Recovered children are at a higher risk of acute malnutrition than children who have never been treated for acute malnutrition (20). Hence, identifying factors associated with relapses of acute malnutrition significantly contributes to achieving the national goal of ending all forms of malnutrition in Ethiopia by 2030 (24). However, relapse following treatment of severe acute malnutrition has rarely been measured across programs and research (23). Therefore, this study aimed to assess the prevalence of acute malnutrition relapse and its associated factors among 6–59 month-old children treated in the community-based acute malnutrition management program in Dessie, Kombolcha, and Haik towns.

## 2 Methods

### 2.1 Study design and period

A community-based cross-sectional study was conducted in Dessie, Kombolcha, and Hiak towns from April 15, 2021, to May 14, 2021.

### 2.2 Study area and setting

This study was conducted in Dessie, Kombolcha, and Haik towns. These three towns are found in the South Wollo zone and are located 401, 373, and 426 km away from Addis Ababa, respectively. According to the Ethiopian Statistics Service 2022 population projection report, Dessie, Kombolcha, and Haik towns have a total of 431,004 residents, of whom 214,829 are men and 216,175 are women. Thirteen health centers in these towns (eight in Dessie city administration, four in Kombolcha town, and one in Haik town) deliver outpatient acute malnutrition management (CMAM) services.

### 2.3 Source and study population

#### 2.3.1 Source population

All children who were treated in the CMAM program in Dessie, Kombolcha, and Haik towns.

#### 2.3.2 Study population

All children aged 6 to 59 months who were treated in the CMAM program and discharged 4 to 54 weeks before data collection.

### 2.4 Eligibility criteria

#### 2.4.1 Exclusion criteria

Children who had treatment outcomes other than recovery (defaulters, lost to follow-up, transfer out), and those with insufficient address information were excluded from the study.

### 2.5 Sample size and sampling procedure

The study included all (356) children who were treated for acute malnutrition in Dessie, Kombolcha, and Haik town health centers and were discharged within 4–54 weeks before the survey. Regarding the sampling procedure, the address information (Kebele, Ketene, name and sex of the child, name of the mother, and phone number) and baseline information about the treatment were obtained from the CMAM logbook. Health extension workers approached mother/caregiver at home. Confirmation of the study subjects was done by asking for the name, age, and sex of the child. Furthermore, the mother/caregiver confirmed that the child was enrolled in the CMAM program at a specified health facility.

### 2.6 Study variables

#### 2.6.1 Dependent variable

Acute malnutrition relapse.

#### 2.6.2 Independent variables

Socio-demographic characteristics: age of the mother, age of the child, education level of the mother, education level of the father, residence, occupation of the mother, and wealth index.

Maternal reproductive health related factors: antenatal care (ANC) follow-up, place of delivery, age of the mother at marriage, family planning, number of children, birth interval, family size and multiple birth.

Child health related factors: diarrhea after management, fever after management, cough after management, chronic diseases, taking anti-helminthic drug, taking Vitamin A and immunization.

Environmental factors: distance from water source, hand hygiene, separate kitchen, and waste disposal.

Child and mother nutrition related factors: taking extra meals during pregnancy, breastfeeding within 1 hour, colostrum feeding, prelacteal feeding, exclusive breast feeding, frequency of breast feeding, separate complementary food preparation, food preparation training, and food aid.

Management related factors: duration of management, MUAC at discharge, WHZ at discharge, and duration (weeks) after discharge.

### 2.7 Operational definition/definition of terms

Acute malnutrition: MUAC <12.5 cm and/or WHZ <−2.

Severe acute malnutrition: MUAC <11.5 cm and/or presence of bilateral edema and/or WFL/H < −3, no medical complication and pass appetite test.

Moderate acute malnutrition: WHZ ≥ −3 Z-score to <−2 Z-score or MUAC ≥ 11.5 cm to <12.5 cm and no edema of both feet.

Relapse: MUAC <12.5 cm (both SAM and MAM) and/or bilateral edema and/or WHZ < −2 following discharge from the CMAM program.

Protected water source: water source from the pipe or modern well.

Food preparation training: mothers/caretakers who have received training in food preparation for children from health professionals, health extension workers, etc.

Wealth index: it was categorized into four quintiles (five groups), namely: poorest, poor, medium, rich, and richest, based on principal component analysis (PCA).

### 2.8 Data collection tools and techniques

Data were collected using a semi-structured questionnaire designed after reviewing related articles (21–23, 25–37). Information related to the treatment and address of the participants was collected from the malnutrition registration logbook and medical records. After obtaining the address information, health extension workers accessed the participants and recorded the child’s current nutritional status and other related data. The weight of the children was measured using a well-calibrated, portable digital weight scale. A portable stadiometer was used to measure the height of the child, and an infantometer was used to measure the length of children under the age of two. MUAC of the child was measured using non-stretchable tape snugly at the midpoint of the arm. The wealth index was computed using fifteen items from the standard equity tool (38). The data were collected by nine trained health extension workers for four weeks and supervised by three MSc pediatric nursing professionals.

### 2.9 Data quality assurance

Data quality was ensured through the training of data collectors, regular supervision, immediate feedback, and spot-checking. Moreover, supervisors and principal investigators checked the completeness of the collected data daily. Pretesting of the questionnaire was undertaken on 5% (16) of the final sample size in Kutaber town.

### 2.10 Data processing and analysis

All filled questionnaires were checked for completeness, consistency, and accuracy. The data were then entered into EPI data (version 4.4.1.0) and exported to SPSS version 25 software for analysis. Descriptive statistics (frequency table, pie chart, and bar graph) were performed to summarize the data. WHZ were generated using WHO Anthro software 3.2.2. Bivariate logistic regression was used to check variables associated with acute malnutrition relapse. Variables found to have a *p*-value ≤0.2 were further analyzed using multivariate logistic regression. Crude and adjusted odds ratio with 95% CI were computed, and variables that had a *p*-value <0.05 in the multivariate logistic regression were considered as significantly associated with the dependent variable. Furthermore, the model fitness was checked using the Hosmer and Lemeshow goodness-of-fit test, which gave a *p*-value of 0.28.

## 3 Result

### 3.1 Socio-demographic characteristics of the respondents

**Figure 1:**
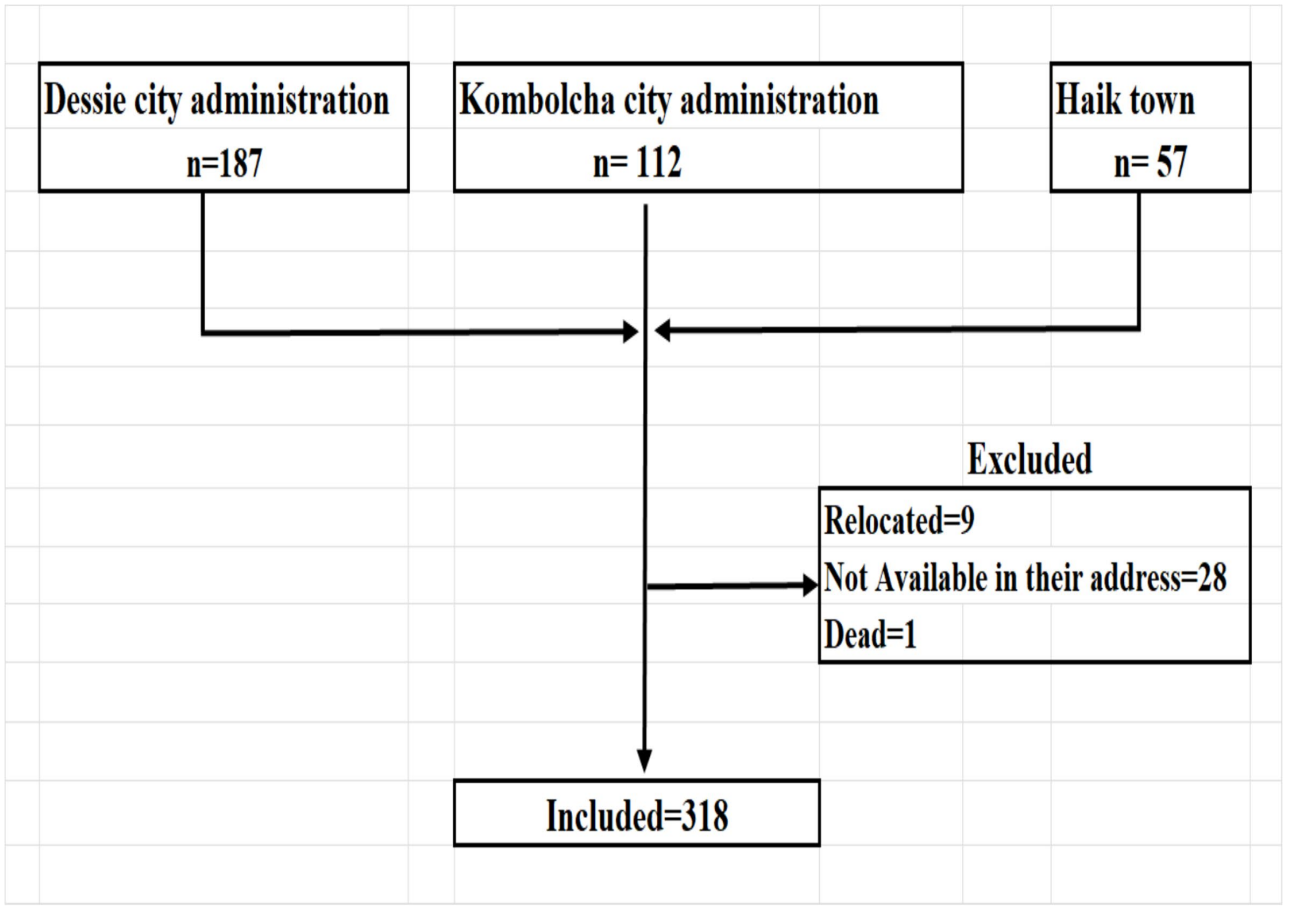
Flow diagram for inclusion of subjects in the analysis (*N* = 318).

In the study areas, there were a total of 356 children treated for acute malnutrition and discharged within 4–54 weeks before the survey. Among those treated children, 318 were accessed and included in the analysis (Figure 1). The majority (59.1%) of mothers/caretakers were aged 25–34 years, with a mean ± SD of 30 ± 5.5 years. Over half (54.4%) of children were male and 49.7% were aged ≥24 months, with a mean ± SD of 27.9 ± 14 months. Nearly three-fourths (72.6%) of mothers/caretakers were housewives, 89.3% were married, 7.2% had a college or university education, and 88.7% were urban dwellers. Nearly one-third (31.4%) of fathers were daily laborers, 34% had primary education, and 22.3% of families had the lowest wealth index (Table 1).

**Table 1:** Socio-demographic characteristics of the study participants in Dessie, Kombolcha, and Haik towns, Northeast Ethiopia (*N* = 318).

Variable	Categories	Frequency (*n*)	Percent (%)
Age of the mother	< 25 years	54	17.0
	25–34Years	188	59.1
	≥35 years	76	23.9
Age of the child	<24 months	160	50.3
	≥24 months	158	49.7
Sex of the child	Male	173	54.4
	Female	145	45.6
Education level of the mother/caretaker	Unable to read and write	38	11.9
	Able to read and write	84	26.4
	Primary school	122	38.4
	Secondary school	51	16.0
	College/University	23	7.2
Education level of the father	Unable to read and write	28	8.8
	Able to read and write	67	21.1
	Primary school	108	34.0
	Secondary school	75	23.6
	College/University	33	10.4
	Missing	7	2.2
Marital status	Married	284	89.3
	Widowed	12	3.8
	Divorced	22	6.9
Residence	Urban	282	88.7
	Rural	36	11.3
Occupation of the mother/caretaker	Housewife	231	72.6
	Government employee	32	10.1
	Farmer	12	3.8
	Merchant	6	1.9
	Daily laborer	37	11.6
Occupation of the father	Government employee	83	26.1
	Private employee	45	14.2
	Farmer	47	14.8
	Merchant	26	8.2
	Daily laborer	100	31.4
	Other	10	3.1
	Missing	7	2.2
Wealth Index	Poorest	65	20.4
	Poor	66	20.8
	Medium	60	18.9
	Rich	65	20.4
	Richest	62	19.5

### 3.2 Child and mother/caretaker-related factors

The vast majority (91.2%) of mothers attended ANC, 94% delivered in health facilities, and 72.6% utilized family planning at the time of data collection. One-fourth (23.9%) of children had diarrhea, 24.2% had fever, and 27.4% had cough after malnutrition treatment. Almost all children (98.1%) had no chronic disease, 31.1% took anthelmintic drugs, 87.7% took vitamin A, and 84.3% were immunized according to the schedule. The birth interval for half of the mothers (49.7%) was more than 2 years, 39.3% of the households had five people, and 97.2% of the deliveries were singletons. A quarter of the households (23%) had a round-trip of more than 30 min to fetch water, 85.2% had a separate kitchen, and 9.4% disposed waste in an open field (Table 2).

**Table 2:** Participant and environmental -related characteristics in Dessie, Kombolcha, and Haik towns, Northeast Ethiopia (*N* = 318).

Variable	Categories	Frequency (*n*)	Percent (%)
Is the mother alive	Yes	312	98.1
	No	6	1.9
Is the father alive	Yes	311	97.8
	No	7	2.2
Did the mother attend ANC	Yes	290	91.2
	No	28	8.8
Number of ANC visits	One	4	1.4
	Two	5	1.7
	Three	75	25.9
	≥ Four	206	71.0
Place of delivery	Health facility	299	94.0
	Home	19	6.0
Age of the mother at marriage	<18 years	23	7.2
	≥18 years	295	92.8
Currently on family planning	Yes	231	72.6
	No	87	27.4
Diarrhea after malnutrition management	Yes	76	23.9
	No	242	76.1
Fever after malnutrition management	Yes	77	24.2
	No	241	75.8
Cough after malnutrition management	Yes	87	27.4
	No	231	72.6
Did the child have chronic disease	Yes	6	1.9
	No	312	98.1
Did the child take an anti-helminthic drug	Yes	99	31.1
	No/not eligible	219	68.9
Did the child take Vitamin A	Yes	279	87.7
	No	39	12.3
Did the child take vaccine	Yes, up to date	268	84.3
	Partial	30	9.4
	Didn’t took	20	6.3
Number of children	≤2 children	200	62.9
	>2 children	118	37.1
Birth interval	First child	92	28.9
	<2 years	68	21.4
	≥2 years	158	49.7
Family size	<5 person	193	60.7
	≥5 person	125	39.3
Was the delivery multiple	Yes	9	2.8
	No	309	97.2
The round trip distance to fetch water	≤30 min	245	77.0
	>30 min	73	23.0
When did you wash your hand (multiple select)	Before feeding	305	95.9
	Before cooking	302	95.0
	After keeping a child’s hygiene	300	94.3
	Before feeding the child	283	89.0
	Before breastfeeding	238	74.8
	After excretion	301	94.7
Kitchen status with the dwelling	Separated	271	85.2
	Not separated	47	14.8
Dry waste disposal system	Disposal pit	125	39.3
	Open field	30	9.4
	Modern method	163	51.3

### 3.3 Nutritional factors

**Figure 2:**
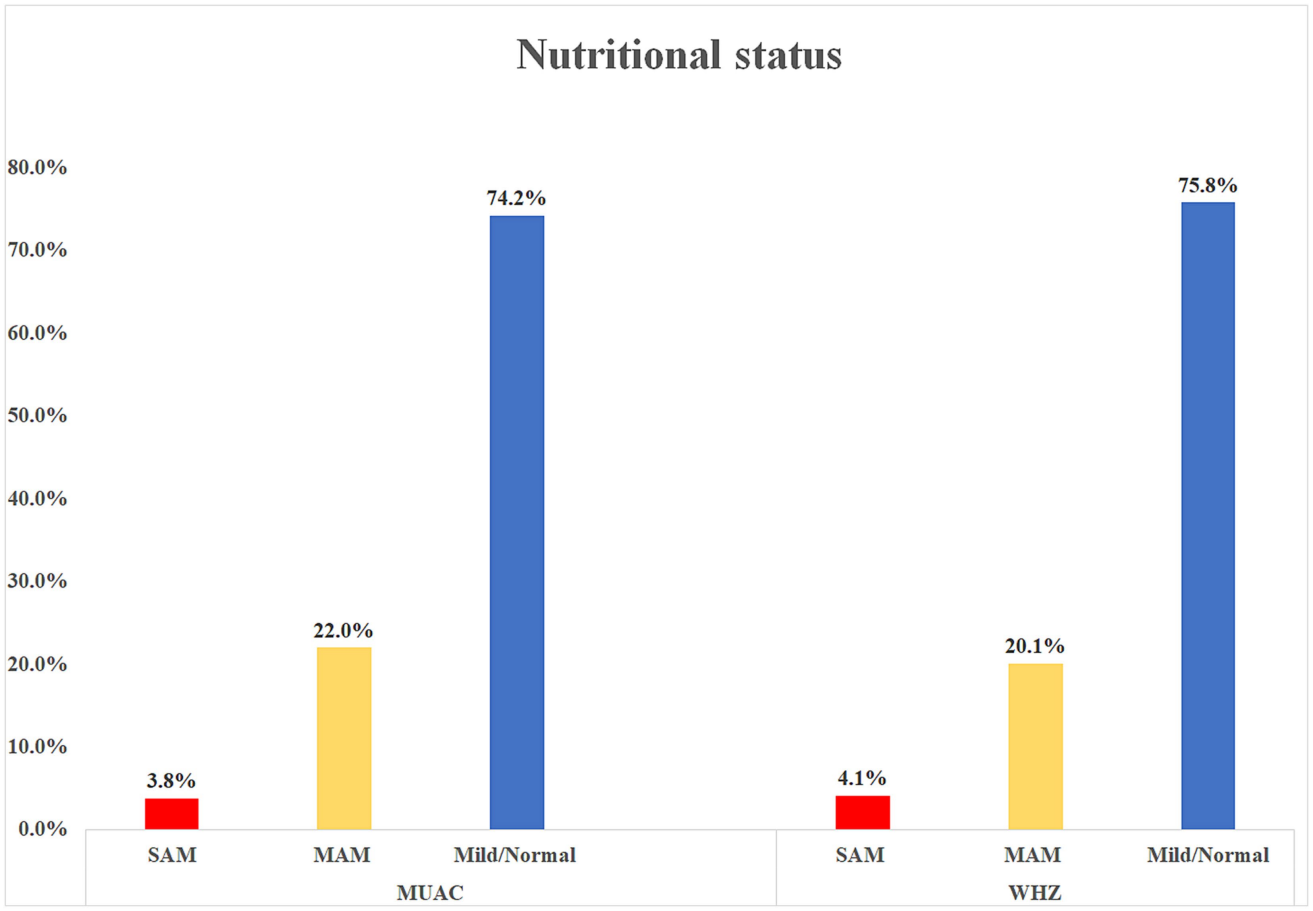
Nutritional status of children using MUAC and WHZ measurements in Dessie, Kombolcha, and Haik towns, Northeast Ethiopia (*N* = 318).

**Figure 3:**
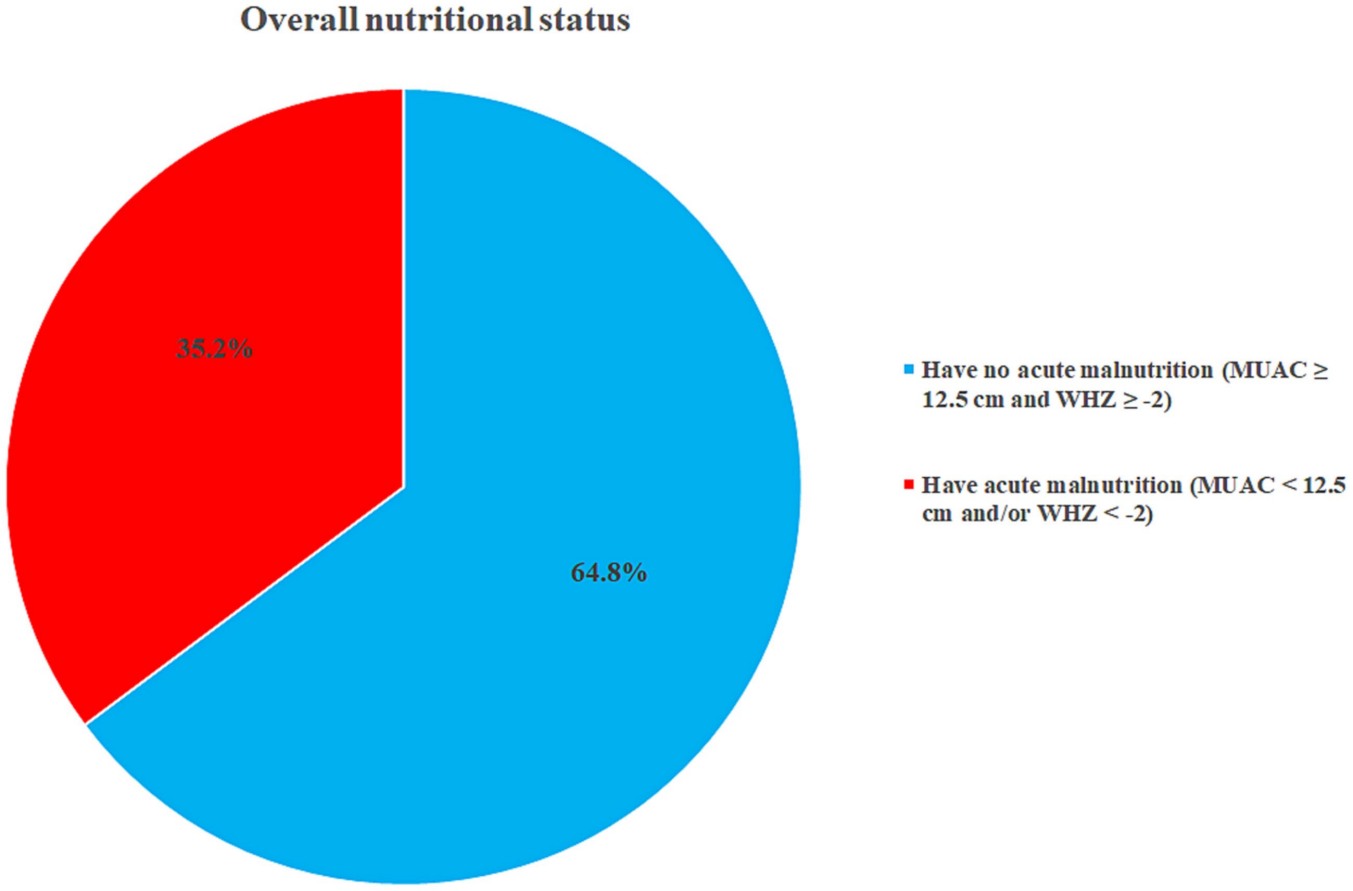
Overall nutritional status of children in Dessie, Kombolcha, and Haik towns, Northeast Ethiopia (*N* = 318).

One-fourth (26.7%) of mothers ate extra meals while pregnant, 88.1% of children received colostrum, 91.2% exclusively breastfed for 6 months, and 84% of mothers/caregivers prepared food separately for the child. Almost one-tenth of the children (10.7%) received malnutrition treatment for 4 weeks, 26.1% had MUAC measurements of 13 cm, and 89.6% had a WHZ of −2 at discharge (Table 3). During the assessment, 3.8% of children had a MUAC measurement of <11.5 cm, 4.1% had a WHZ score of <−3, and 35.2% (95% CI; 29.9, 40.6%) of children had acute malnutrition (Figures 2, 3).

**Table 3:** Nutritional characteristics of the study participants in Dessie, Kombolcha, and Haik towns, Northeast Ethiopia (*N* = 318).

Variable	Categories	Frequency (*N*)	Percent (%)
Did the mother take extra meals during pregnancy	Yes	85	26.7
	No	233	73.3
Breastfeeding within 1 hour of delivery	Yes	266	83.6
	No	52	16.4
Does the child feed Colostrum	Yes	280	88.1
	No	38	11.9
Was their prelacteal feeding	Yes	25	7.9
	No	293	92.1
Exclusive breastfeeding up to 6 months	Yes	290	91.2
	No	28	8.8
Start complementary feeding after 6 months	Yes	300	94.3
	No	18	5.7
Currently on breastfeed	Yes	155	48.7
	No	163	51.3
How many times a day does/did the child feed	≥ 8 times	194	61.0
	< 8 times	124	39.0
Prepare food for the child separately	Yes	267	84.0
	No	51	16.0
Did you get education about food preparation for the child	Yes	50	15.7
	No	268	84.3
Is the HH currently on food aid	Yes	46	14.5
	No	272	85.5
Duration of treatment	≤ 4 weeks	34	10.7
	5−12 weeks	216	67.9
	>12 weeks	68	21.4
MUAC at discharge	<13 cm	235	73.9
	≥ 13 cm	83	26.1
WHZ at discharge	≥ − 2	285	89.6
	<−2	33	10.4
Duration after discharge	4–27 weeks	204	64.2
	27–54 weeks	114	35.8

### 3.4 Factors associated with acute malnutrition relapse

In the bivariate logistic regression, the age of the child, educational level of the mother, diarrhea after management, immunization status, birth space, MUAC at discharge, WHZ at discharge, duration after discharge, and wealth index had a *p*-value of ≤0.2. Further analysis of these variables in the multivariable logistic regression revealed that age of the child, immunization status, diarrhea after management, MUAC at discharge, and wealth index had a significant association with acute malnutrition relapse.

Children aged <24 months had higher odds of acute malnutrition relapse compared to ≥24 months-old children (AOR: 3.08, 95% CI; 1.76, 5.39). Likewise, children who had diarrhea after management were more likely to experience malnutrition relapse than those who did not (AOR: 2.93, 95% CI; 1.51, 5.69). Additionally, children who were not immunized had higher odds of acute malnutrition relapse than those children with full immunization (AOR: 3.05, 95% CI; 1.14, 8.23). Furthermore, children with a discharge MUAC of <13 cm had a higher risk of acute malnutrition relapse than those with a MUAC of ≥13 cm (AOR: 3.16, 95% CI: 1.56, 6.40). Finally, children from the poorest and poor families had higher odds of developing acute malnutrition relapse compared to children from the richest families [(AOR: 3.65, 95% CI; 1.45, 9.18) and (AOR: 2.73, 95% CI; 1.13, 6.59), respectively] (Table 4).

**Table 4:** Factors associated with acute malnutrition relapse in Dessie, Kombolcha, and Haik towns, Northeast Ethiopia (*N* = 318).

Variables	Category	Acute malnutrition		COR (95% CI)	AOR (95% CI)	*p*-value
		Yes	No			
Age of the child	<24 months	75 (46.9%)	85 (53.1%)	2.89 (1.78, 4.67)	3.08 (1.76, 5.39)	**<0.001**
	≥24 months	37 (23.4%)	121 (76.6%)	1		
The educational level of the mother	Unable to read and write	18 (47.4%)	20 (52.6%)	2.551 (0.83, 7.88)	2.78 (0.76, 10.17)	0.12
	Able to read and write	29 (34.5%)	55 (65.5%)	1.49 (0.53, 4.20)	2.69 (0.79, 9.13)	0.11
	Primary education	43 (35.2%)	79 (64.8%)	1.54 (0.57, 4.20)	2.61 (0.82, 8.38)	0.11
	Secondary education	16 (31.4%)	35 (68.6%)	1.30 (0.43, 3.90)	1.77 (0.52, 6.029)	0.36
	College/university	6 (26.1%)	17 (73.9%)	1	1	
Diarrhea after treatment	Yes	34 (44.7%)	42 (55.3%)	1.70 (1.01, 2.88)	2.93 (1.51, 5.69)	**0.001**
	No	78 (36.8%)	164 (63.2%)	1	1	
Immunization	Yes, up-to-date	91 (34%)	177 (66.0%)	1	1	0.68
	Partial	10 (33.3%)	20 (66.7%)	0.97 (0.44, 2.17)	0.81 (0.31, 2.16)	**0.03**
	Didn’t take	11 (55.0%)	9 (45.0%)	2.38 (0.95, 5.94)	3.05 (1.14, 8.23)	
Birth interval	First child	32 (34.8%)	60 (65.2%)	1.19 (0.69, 2.05)	1.00 (0.52, 1.95)	0.99
	< 2 years	31 (45.6%)	37 (54.4%)	1.86 (1.04, 3.34)	1.11 (0.53, 2.30)	0.78
	≥ 2 years	49 (31.0%)	109 (69.0%)	1	1	
MUAC at discharge	<13 cm	96 (40.9%)	139(59.1%)	2.89 (1.58, 5.29)	3.16 (1.56, 6.40)	**0.001**
	≥13 cm	16 (19.3%)	67 (80.7%)	1	1	
WHZ at discharge	<−2	16(48.5%)	17 (51.5%)	1.85 (0.90, 3.83)	1.66 (0.71, 3.88)	0.24
	≥ − 2	96 (33.7%)	189 (66.3%)	1	1	
Duration after discharge	4–27 weeks	83 (40.7%)	121 (59.3%)	2.01 (1.21, 3.33)	1.26 (0.68, 2.31)	0.46
	27–54 weeks	29 (25.4%)	85 (74.6%)	1	1	
Wealth index	Poorest	36 (55.4%)	29 (44.6%)	2.13 (1.02, 4.48)	3.65 (1.45, 9.18)	**0.006**
	Poor	41 (62.1%)	25 (37.9%)	1.61 (0.76, 3.41)	2.73 (1.13, 6.59)	**0.03**
	Medium	41 (68.3%)	19 (31.7%)	1.23 (0.56, 2.67)	2.14 (0.84, 5.45)	0.11
	Rich	43 (66.2%)	22 (33.8%)	1.35 (0.63, 2.89)	1.16 (0.47, 2.82)	0.75
	Richest	45 (72.6%)	17 (27.4%)	1	1	

## 4 Discussion

According to the findings of this study, the prevalence of overall acute malnutrition relapse among children treated in the CMAM program was 35.2% (95% CI, 30.2, 41.5%). 6.6% of the relapsed cases were SAM and 28.6% were MAM, indicating that more than one-fourth of the treated children had returned to MAM. Other studies conducted in Gambella region and Hadiya zone indicated a 10.1 and 9.6% relapse to SAM, respectively which is higher than the relapse reported in this study (6.6%) (39, 40). The discrepancy might be attributed to variations in the study design, setting and cultural and sociodemographic diferences. On the other side, comparable findings of overall acute malnutrition relapse were reported from studies conducted in Malawi (41%), Eastern Ethiopia (36.2%) and South Gondar, Ethiopia (34.3%) (11, 20, 41). However, studies in Burkina Faso (15.4%), a systematic review of low and middle-income countries (27.5%), and the United States (27%), all found a lower prevalence of relapse (22, 28, 42). To the contrary, a higher magnitude of relapse was reported from the study in the Democratic Republic of the Congo (44.2%) (43). These discrepancies might be attributed to the variations in the study design, duration of follow-up, study setting, and cultural and economic factors.

One of the significantly associated variables with the relapse of acute malnutrition was the child’s age. Children aged <24 months had higher odds of acute malnutrition relapse compared to children older than ≥24 months. Children under the age of two require more nutrition and energy for growth and development than at any other stage in their lives, making them more vulnerable to malnutrition (44). Similar studies from Nepal and Nigeria also reported that the age of children had an association with malnutrition (45, 46).

In this study, having diarrhea after discharge from malnutrition management was significantly associated with acute malnutrition relapse. Children who had diarrhea after discharge had higher odds of acute malnutrition relapse compared to children without diarrhea. Diarrhea contributes to malnutrition by reducing food intake, decreasing nutrient absorption, and increasing nutrient catabolism (47). This finding is consistent with other studies undertaken in Ethiopia (48) and Nigeria (49).

The immunization status of children had a significant association with acute malnutrition relapse. Children who were not immunized experienced a higher occurrence of acute malnutrition relapse compared to fully vaccinated children. Immunization protects children from a wide range of illnesses, including pneumonia and diarrhea, which can put them at risk of malnutrition. A significantly higher prevalence of malnutrition was found among children with incomplete vaccinations (50). Research findings in this regard illustrate the relationship between vaccination and nutritional status (50, 51).

Furthermore, the MUAC measurement of children at discharge showed significant associations with acute malnutrition relapse. Children with a MUAC measurement of <13 cm were more likely to have an acute malnutrition relapse compared to children with a MUAC measurement of ≥13 cm. Adequate nutritional achievement before discharge might enable the child to sustain their nutritional status. A similar study from Ethiopia and Nigeria also reported that the discharge MUAC of children was a significant predictor of relapse (40, 46).

Finally, the wealth index was reported to have a significant association with acute malnutrition relapse. Children from the poorest families had higher odds of developing an acute malnutrition relapse compared to children from the richest families. Poverty and malnutrition are deeply interrelated, and low socioeconomic status remains one of the most important predictors of child malnutrition (52). Related studies from India (53), Ethiopia (27, 54), and Bangladesh (55) reported similar results.

### 4.1 Limitations of the study

This study addresses the gross acute malnutrition relapses from 1 to 12 months following CMAM treatment. It did not figure out the relapse rate in relation to various periods. Therefore, further large-scale prospective cohort studies are needed to describe the relapse rate in various periods. Moreover, given that the study used a cross-sectional design, the associations observed in the study do not infer a causal relationship, and there might be recall biases for certain items.

## 5 Conclusion

Over one-third of children treated under the CMAM program reverted to SAM or MAM. The age of the child, diarrhea after discharge, immunization, MUAC at discharge, and wealth index were all significantly associated variables with acute malnutrition relapse. Therefore, adequate health education and counseling services are required for mothers to improve child immunization coverage and maintain adequate hygiene to prevent diarrhea. In addition, further experimental research is needed to investigate the effect of MUAC at discharge on the risk of acute malnutrition relapse.

## Data availability statement

The raw data supporting the conclusions of this article will be made available by the authors, without undue reservation.

## Ethics statement

Ethical clearance and approval (RF: CMHS/47/13/13) were obtained from Wollo University College of Medicine and Health Sciences. Official letters were written to the respective health facilities and town administrations. Consent to review the medical records of study subjects was waived by the authorities of health facilities. Written informed consent was obtained from the mother/caretaker after thoroughly explaining the objectives of the study and the data were analyzed anonymously. Respondents had the right to not participate in or withdraw from the study at any stage, and compliance with the Declaration of Helsinki was ensured.

## Author contributions

YY: Conceptualization, Data curation, Formal analysis, Funding acquisition, Investigation, Methodology, Project administration, Resources, Software, Supervision, Validation, Visualization, Writing – original draft, Writing – review & editing. ZY: Investigation, Project administration, Software, Supervision, Validation, Visualization, Writing – review & editing. SN: Data curation, Investigation, Project administration, Supervision, Validation, Visualization, Writing – review & editing. DJ: Formal analysis, Investigation, Software, Supervision, Validation, Writing – review & editing.
